# Supplementary material for: Marketing of medicines in primary care: An analysis of direct marketing mailings and advertisements
Source: PLoS One. 2023 Aug 28;18(8):e0290603. doi: 10.1371/journal.pone.0290603 (PMC10461816; doi:10.1371/journal.pone.0290603)
Supplement: S1 Table — IUD: Intrauterine device. athe year of registration refers to the year of registration for the marketed indication. If multiple indications were marketed, the first year was mentioned. bthe marketing materials for allergen extracts were for different patented products and have therefore different years of introduction. cthe marketing materials for levonorgestrel were for different dosage forms and have therefore different years of introduction. dthe marketing materials for testosterone were for different dosages and dosage forms and have therefore different years of introduction. (DOCX) [file pone.0290603.s001.docx]

**S1 Table: Characteristics of marketed medicines.**

| **Medicine** | **Medicine group** | **Number of materials** | **Year of marketing approval^a^** |
| --- | --- | --- | --- |
| Allergen extracts^b^ | Allergen extracts | 1 | 2003 |
| Allergen extracts^b^ | Allergen extracts | 1 | 2006 |
| Apixaban | Direct factor Xa inhibitors | 2 | 2011 |
| Bempedoic acid | Other lipid modifying agents | 1 | 2020 |
| Benralizumab | Other systemic drugs for obstructive airway diseases | 1 | 2018 |
| Bimekizumab | Interleukin inhibitors | 1 | 2021 |
| C1-inhibitor, plasma derived | Drugs used in hereditary angioedema | 1 | 2011 |
| Clobetasol | Corticosteroids, very potent (group IV) | 1 | 2004 |
| Dapagliflozin | Sodium-glucose co-transporter 2 (SGLT2) inhibitors | 2 | 2012 |
| Denosumab | Other drugs affecting bone structure and mineralization | 1 | 2010 |
| Dexamethasone (ocular) | Corticosteroids, plain | 1 | 2010 |
| Dexamfetamine | Centrally acting sympathomimetics | 1 | 2021 |
| Dulaglutide | Glucagon-like peptide-1 (GLP-1) analogues | 1 | 2014 |
| Edoxaban | Direct factor Xa inhibitors | 1 | 2015 |
| Empagliflozin | Sodium-glucose co-transporter 2 (SGLT2) inhibitors | 2 | 2014 |
| Emtricitabine, tenofovir alafenamide and bictegravir | Antivirals for treatment of HIV infections, combinations | 1 | 2018 |
| Filgotinib | Selective immunosuppressants | 1 | 2020 |
| Finerenone | Aldosterone antagonists | 3 | 2022 |
| Formoterol and beclometasone | Adrenergics in combination with corticosteroids or other drugs, excl. anticholinergics | 1 | 2007 |
| Formoterol, glycopyrronium bromide and beclometasone | Adrenergics in combination with anticholinergics incl. triple combinations with corticosteroids | 1 | 2017 |
| Formoterol, glycopyrronium bromide and budesonide | Adrenergics in combination with anticholinergics incl. triple combinations with corticosteroids | 1 | 2020 |
| Hydrocortisone | Glucocorticoids | 1 | 2011 |
| Liraglutide | Glucagon-like peptide-1 (GLP-1) analogues | 3 | 2015 |
| Lisdexamfetamine | Centrally acting sympathomimetics | 1 | 2019 |
| Medroxyprogesterone | Progestogens | 2 | 2011 |
| Methenamine | Other antibacterials | 1 | 1966 |
| Naloxegol | Peripheral opioid receptor antagonists | 1 | 2014 |
| Norelgestromin and ethinylestradiol | Progestogens and estrogens, fixed combinations | 1 | 2002 |
| Norethisterone | Estren derivatives | 1 | 1990 |
| Ofatumumab | Selective immunosuppressants | 1 | 2021 |
| Plastic IUD with progestogen^c^ | Intrauterine contraceptives | 1 | 2021 |
| Plastic IUD with progestogen^c^ | Intrauterine contraceptives | 1 | 1996 |
| Rivaroxaban | Direct factor Xa inhibitors | 9 | 2008 |
| Semaglutide | Glucagon-like peptide-1 (GLP-1) analogues | 1 | 2020 |
| Testosterone^d^ | 3-oxoandrosten (4) derivatives | 1 | 2016 |
| Testosterone^d^ | 3-oxoandrosten (4) derivatives | 1 | 2005 |
| Testosterone^d^ | 3-oxoandrosten (4) derivatives | 1 | 2002 |
| Tildrakizumab | Interleukin inhibitors | 1 | 2018 |
| Tiotropium bromide | Anticholinergics | 1 | 2001 |
| Upadacitinib | Selective immunosuppressants | 1 | 2019 |
| Vilanterol, umeclidinium bromide and fluticasone furoate | Adrenergics in combination with anticholinergics incl. triple combinations with corticosteroids | 1 | 2017 |

IUD: intrauterine device.

^a^the year of registration refers to the year of registration for the marketed indication. If multiple indications were marketed, the first year was mentioned.

^b^the marketing materials for allergen extracts were for different patented products and have therefore different years of introduction.

^c^the marketing materials for levonorgestrel were for different dosage forms and have therefore different years of introduction.

^d^the marketing materials for testosterone were for different dosages and dosage forms and have therefore different years of introduction.
